# Editorial: New addictions in the era of digitalization

**DOI:** 10.3389/fnhum.2023.1302635

**Published:** 2023-10-12

**Authors:** Gergely Feher, Antal Tibold, Graca Esgalhado

**Affiliations:** ^1^Centre for Occupational Medicine, Medical School, University of Pécs, Pécs, Hungary; ^2^Department of Psychology and Education, Faculty of Social and Human Sciences, University of Beira Interior, Pólo IV, Covilhã, Portugal

**Keywords:** internet, addiction, editorial, special issue, digitalization and e-health

In addition to many positive effects of the rise of digitization, its dark sides have also been recognized such as problematic usage of the internet (PUI), smartphone addiction or no-mobile phobia (nomophobia). In 2020, the World Health Organization (WHO) formally recognized and labeled digital device addiction as a widespread problem as online activities and compulsive internet use (or problematic usage of the internet) can affect a significant proportion of the whole population worldwide (Dresp-Langley and Hutt, 2022). A recent meta analysis reported an increased rate of digital addiction in the last two decades, pooled prevalence estimates were ~27% for smartphone addiction, 17.5% for social media addiction, 14% for problematic usage of the internet, 8% for cybersex addiction, and 6% for problematic gaming (Meng et al., 2022).

Probably PUI is the most extensively studied digital addiction of our century. It is rather an umbrella term than a single diagnosis and it is still a matter of debate whether general internet addiction is different from its subtypes (if these are subtypes or they are different entities) as seen above (Fineberg et al., 2022).

Several demographic and socioeconomic factors such as male gender, younger age at the onset of digital device use, daily time interval and geographical differences (more common is East-Asia) seem to be predecessors of the phenomena as well as (the lack of) parental control or family conflicts (poor children-parents relationships) but results are inconclusive (Toth et al., 2021; Fineberg et al., 2022). Several personality traits are also strongly related to the phenomena (Fineberg et al., 2022).

In their interesting study Wei et al. examined the role of zhongyong thinking on internet addiction including a large number of participants. Zhongyong thinking coversmulti-thinking (consider things from different perspectives), holism (ability to coordinate with others) and harmoniousness (tendency to avoid potential conflicts), which seems to be protective against PUI through mediating maladaptive cognition (Wu and Lin, 2005).

Boredom can easily be alleviated by digital devices. Boredom susceptibility is a relatively rarely studied personality trait, which can be a risk factor for compulsive digital device use. A recent meta analysis confirmed the role of boredom on general internet use, but it also can be related to PUI, however, future studies are needed (Camerini et al., 2023). Cannito et al. studied the above mentioned association with the inclusion of 69 young adults using a dot-probe task assessing internet-related attentional bias and questionnaires detecting potential PUI and susceptibility to boredom. Based on their results the tendency to experience boredom (and also the selective attention toward social network information) was strongly related to PUI highlighting the potential role of selective attentional processing at the background of compulsive internet use.

PUI seems to be associated with a cluster of mental and physical illnesses such as depression, autism, overweight, eating disorders (Aghasi et al., 2020; Toth et al., 2021; Fineberg et al., 2022). Interestingly, a relatively few studies focused on the association of burnout and PUI, which may share similar risk factors and consequences and they are still labeled as phenomena and not as medical conditions despite intensive research carried out in the last decades (Toth et al., 2021). In their study involving ~2,500 adolescents Feher et al. showed significant associations among PUI, insomnia, depression and burnout after the setup of a logistic regression model.

The predecessors of problematic online behaviors extensively studied, but relatively few studies focused on protective factors such as positive youth development (PYD). In their interesting follow-up study Gan et al. showed the positive effect of PYD on depression, internet gaming disorder and cyberbullying/victimization during the recent pandemic, which may have had a negative impact on digital addictions, but seems to be a transient phenomenon to cope with isolation and psychological distress, however, long-term effects are still unknown (Casale et al., 2023).

Mobile phone addiction is also a well-known digital addiction with extensively growing literature. Probablysocial media applications are the most extensively used functions and mobile social media addiction is one of the main determinants of global mobile addiction (Gao et al., 2022). Peer pressure is a main element of compulsive use and may be mediated by personality traits. In their interesting study Xu et al. showed the significant effect of peer pressure on mobile social media addiction among adolescents, and peer pressure was more likely mediated by self-esteem than self-concept.

Vast majority of digital addictions studies usually recruit adolescents and older individuals are relatively sparsely studied. These populations may have different risk factors and predecessors of compulsive digital use (Ioannidis et al., 2018). In a Chinese study by Xu et al. including 371 adults aged ≥45 years showed that the feeling of cognitive decline and impaired family relationships can be associated with mobile addiction mediated by the feeling of alienation, opening new horizons in understanding the digital addiction of this age group.

Finally, apart from the above mentioned potential harmful effects, the use of digital technologies open new pathways in patient management. Ren et al. showed the efficacy of 5G-based teleultrasound robotic diagnostic system in abdominal scanning, which does not require the presence of radiologist. These technologies can apply in underserved areas widening the accessibility of healthcare services.

## Author contributions

GF: Writing—original draft, Writing—review and editing. AT: Writing—original draft, Writing—review and editing. GE: Writing—original draft, Writing—review and editing.
